# Prolonged survival after small-cell transformation of EGFR-mutant lung adenocarcinoma: a case report

**DOI:** 10.3389/fonc.2026.1975965

**Published:** 2026-10-26

**Authors:** Yaxin Yan, Dongjie Du, Fenghua Wen, Jiaqi Wang, Yitao Jia

**Affiliations:** 1 Department of Oncology, Hebei General Hospital, Shijiazhuang, Hebei, China; 2 Vascular Surgery Catheterization Room, Hebei General Hospital, Shijiazhuang, Hebei, China

**Keywords:** case report, EGFR gene mutation, EGFR-TKI resistance, non-small cell lung cancer, transformed small cell lung cancer

## Abstract

Transformed small cell lung cancer (T-SCLC) is most commonly observed in EGFR-mutant lung adenocarcinoma and represents an important mechanism of acquired resistance to EGFR tyrosine kinase inhibitors (EGFR-TKIs). Its reported clinical incidence is approximately 3%-10%. Patients with T-SCLC often experience rapid disease progression and poor overall prognosis, and no established standard treatment regimen is currently available. We report a case of advanced EGFR-mutant lung adenocarcinoma with *RB1*, *TP53*, and *BRCA2* co-mutations that underwent small-cell transformation during treatment. Small-cell transformation was detected through close surveillance and confirmed by repeat biopsy. The patient subsequently received individualized multimodal therapy, including chemotherapy, targeted therapy, and local radiotherapy, and remained alive 43 months after the initial diagnosis.

## Introduction

Transformed small cell lung cancer (T-SCLC) refers to the histological transformation from non-small cell lung cancer (NSCLC) to small cell lung cancer (SCLC), which is predominantly observed in patients with epidermal growth factor receptor (EGFR)-mutant lung adenocarcinoma and represents one of the important mechanisms of resistance to EGFR-tyrosine kinase inhibitors (EGFR-TKI) (1). The incidence of transformation to SCLC after EGFR-TKI treatment in NSCLC patients is approximately 3%-10% (2), with a median time from lung cancer diagnosis to transformation of 13-18months (3). T-SCLC is more commonly observed in never-smoking young female patients (4). *EGFR* exon 19 deletion (19DEL) is more prone to undergo SCLC transformation compared with exon 21 L858R mutation (L858R) (5). Furthermore, SCLC transformation can also occur in anaplastic lymphoma kinase (ALK)-mutant and c-ros oncogene 1 (ROS1)-mutant lung cancers following targeted therapy, as well as in NSCLC patients receiving chemotherapy or immune checkpoint inhibitors (2, 6–8). Regardless of the etiology, once SCLC transformation occurs, it typically indicates aggressive disease progression and poorer prognosis, with a median overall survival (OS) of only 10.9 months (8–14 months) (4). The authors report a case of an advanced EGFR-mutant lung adenocarcinoma patient who developed SCLC transformation after EGFR-TKI treatment. However, due to timely detection and comprehensive treatment including chemotherapy, targeted therapy, and local radiation therapy, the patient achieved long-term survival of 43 months, which is presented herein.

## Case description

A 54-year-old woman presented in December 2022. She had never smoked and had no significant family history of malignancy. She reported dyspnea for 6 months, with acute worsening over the preceding 10 days.Thoracic CT showed a large right pleural effusion. Multiple focal pleural abnormalities were also present, including thickening, nodules, and masses (**Figure 1A**).Whole-body bone scintigraphy showed increased tracer uptake in the right clavicle and the T10 vertebra. These findings were suspicious for bone metastases. The pleural fluid was hemorrhagic. Cytopathology revealed atypical cells consistent with adenocarcinoma. Immunohistochemistry was positive for TTF-1 and Napsin A, supporting a pulmonary origin. Percutaneous needle biopsy of the right lung mass confirmed invasive adenocarcinoma. The immunohistochemical panel demonstrated: CK7 (3+), TTF-1 (2+), CK5/6 (focal +), Ki-67 (70%+), p63 (focal +), p40 (focal +), Napsin A (3+). These findings are consistent with a primary pulmonary adenocarcinoma of non-small cell type. Next-generation sequencing (NGS) was performed on tissue samples at Simcere Diagnostic Laboratories using a 74-gene panel. The identified mutations included *EGFR L858R* mutation (17.34%), *BRCA2* mutation (4.35%), *RB1* mutation (4.01%), and *TP53* mutation (2.08%), with variant allele frequencies shown in parentheses. Final Diagnosis: Right lower lobe adenocarcinoma with pleural and osseous metastases. TNM Staging: T4N0M1c, Stage IVB according to the 8th edition of AJCC Cancer Staging Manual.

**Figure 1:**
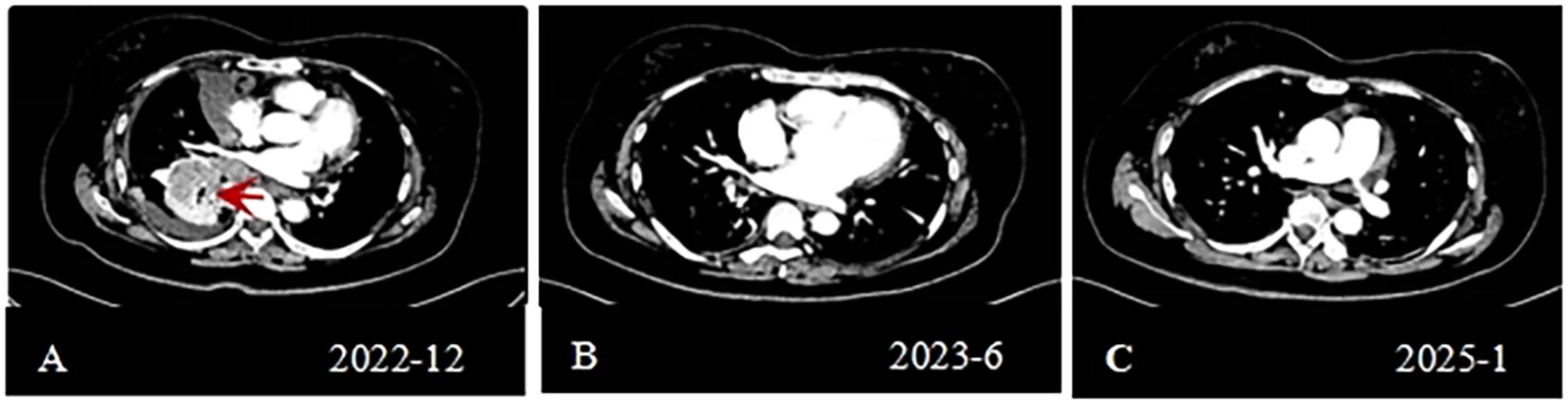
Changes in right pulmonary lesion before and after treatment. **(A)** Right lower lobe lesion (red arrow) and pleural effusion (blue arrow) at initial diagnosis (December 2022). **(B)** Resolution of right pulmonary lesion during maintenance therapy with osimertinib combined with pemetrexed (June 2023). **(C)** Resolution of right pulmonary lesion during maintenance therapy with osimertinib alone (January 2025).

After multidisciplinary team (MDT) discussion, the patient was considered unsuitable for surgery and systemic therapy was planned. While awaiting molecular testing results, she received one cycle of intrapleural recombinant human endostatin (45mg on days 1, 4, and 7) plus cisplatin (40mg on days 1, 4, and 7) to relieve dyspnea and control the right pleural effusion. Following the genetic testing results, from January to March 2023 she received osimertinib (80mg once daily) combined with pemetrexed and carboplatin for 4 cycles, achieving a partial response (PR) according to RECIST 1.1. From April 2023 to March 2024, she received osimertinib plus pemetrexed maintenance therapy for 14 cycles. In September 2023, the patient developed back pain. MRI suggested a thoracic vertebral metastasis (T10). Local radiotherapy was added to alleviate symptoms and reduce fracture risk. The prescribed dose was 95% PTV 30 Gy/10 fractions, 3Gy/fraction. The patient completed radiotherapy between September 25 and October 9, 2023. In March 2024, the patient developed nausea and diarrhea(CTCAE grade 3) and could not tolerate pemetrexed chemotherapy; chemotherapy was stopped and osimertinib monotherapy continued. Ongoing regular monitoring showed disease stability (**Figure 1**).

At follow-up evaluation on March 19, 2025, elevated gastrin-releasing peptide precursor (Pro-GRP) level was documented (**Figure 2**). PET/CT imaging demonstrated newly developed right-sided pleural involvement (**Figure 3A**). Therapeutic response assessment indicated progressive disease (PD) according to RECIST 1.1. On March 21, 2025, ultrasound-guided biopsy of the right-sided pleural lesion was performed. Pathological examination revealed findings consistent with small cell carcinoma. Immunohistochemical staining demonstrated the following profile: CK (1+), TTF-1 (3+), CD56 (3+), Syn (3+), ChrA (2+), p53 (1+), Rb (1+), Ki-67 (70%+), LCA (–), Vimentin (–), ATRX (+), P40 (–), CK5/6 (–) (**Figure 4**). The tissue samples were insufficient. NGS was performed on blood samples at Decode DNA using a 624-gene panel. The identified mutations included *EGFR L858R* mutation (3.52%), *BRCA2* mutation (4.43%), and *TP53* mutation (0.73%), with variant allele frequencies shown in parentheses. After MDT discussion, the patient was diagnosed with T-SCLC. Given the isolated progression of the lesion and the persistent *EGFR L858R* mutation identified on genetic testing, a treatment strategy was adopted comprising continued osimertinib targeted therapy combined with local radiotherapy to the right-sided pleural metastasis. Radiotherapy dose prescription: 60 Gy in 20 fractions (3 Gy per fraction) to 95% planning gross target volume (PGTV). On July 8, 2025, follow-up PET/CT revealed multiple metastatic lesions in hepatic segments S6 and S8 (**Figures 3B, C**), with disease evaluation indicating PD according to RECIST 1.1. On July 10, 2025, ultrasound-guided liver biopsy was performed. Histopathological examination confirmed small cell carcinoma. Immunohistochemistry demonstrated: CK (+), CK19 (2+), TTF-1 (3+), CD56 (2+), Syn (2+), ChrA (2+), p53 (1+), Rb (–), Ki-67 (90%+), SSTR2 (-) (**Figure 5**). NGS was performed on tissue samples at Decode DNA using a 85-gene panel. The identified mutations included *EGFR L858R* mutation (72.72%), *BRCA2* mutation (93.22%), *TP53* mutation (66.3%), *ERBB2* amplification (3.7 copies) and *RB1* mutation (86.35%). The patient was diagnosed with T-SCLC with rapid disease progression. Given the persistent *EGFR L858R* mutation, the treatment regimen was modified based on primary small cell lung cancer protocols. The patient received osimertinib (80mg once daily) targeted therapy combined with etoposide plus carboplatin(EC) chemotherapy. From August 8, 2025 to November 26, 2025, the patient underwent six cycles of etoposide (0.15g on days 1–3, every 21 days) plus carboplatin (0.5g on day 1, every 21 days), with concurrent oral osimertinib (80 mg once daily). After three cycles of treatment, follow-up abdominal non-contrast CT demonstrated partial reduction in some hepatic metastases with complete resolution of others, achieving PR (**Figures 5A–D**). Following completion of six cycles, repeat CT imaging showed continued mild reduction of hepatic metastases, with sustained PR according to RECIST 1.1. After six cycles of EC chemotherapy, the treatment was transitioned to osimertinib combined with anlotinib targeted therapy. Beginning December 25, 2025, the patient received maintenance therapy with anlotinib (8mg once daily on days 1–14, every 21 days) plus osimertinib (80 mg once daily). Follow-up PET/CT in February 2026 demonstrated sustained PR according to RECIST 1.1 (**Figures 5E–G**). As of July 6, 2026, the patient achieved an OS of 43 months, with survival exceeding 12 months following small cell lung cancer transformation (**Figure 6**).

**Figure 2:**
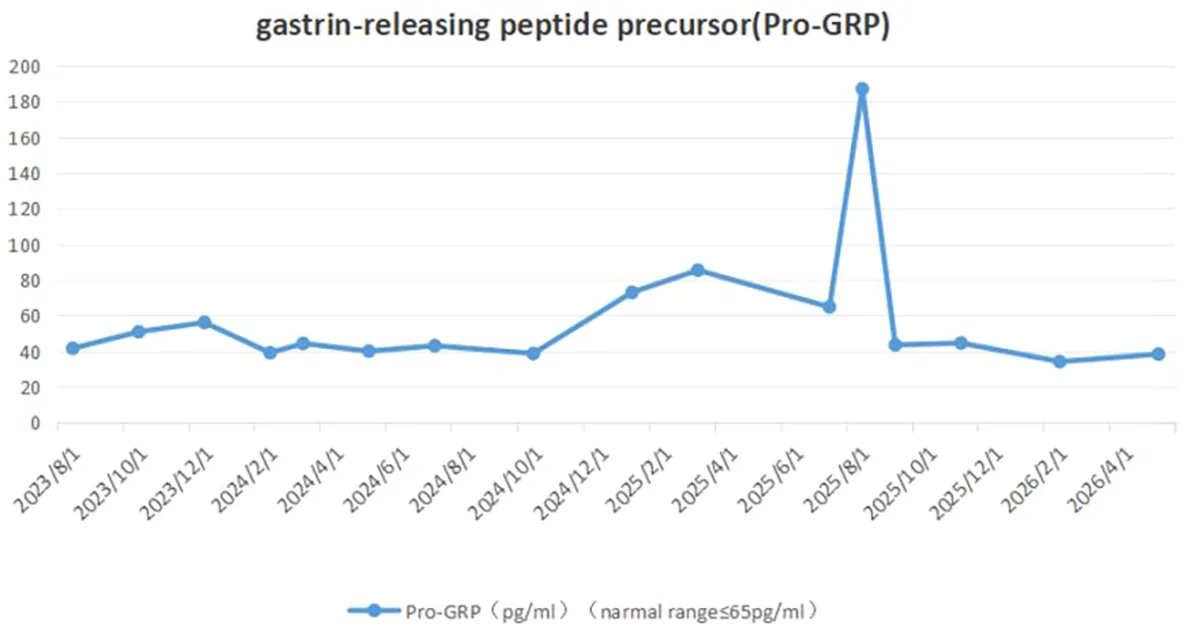
ProGRP level changes.

**Figure 3:**
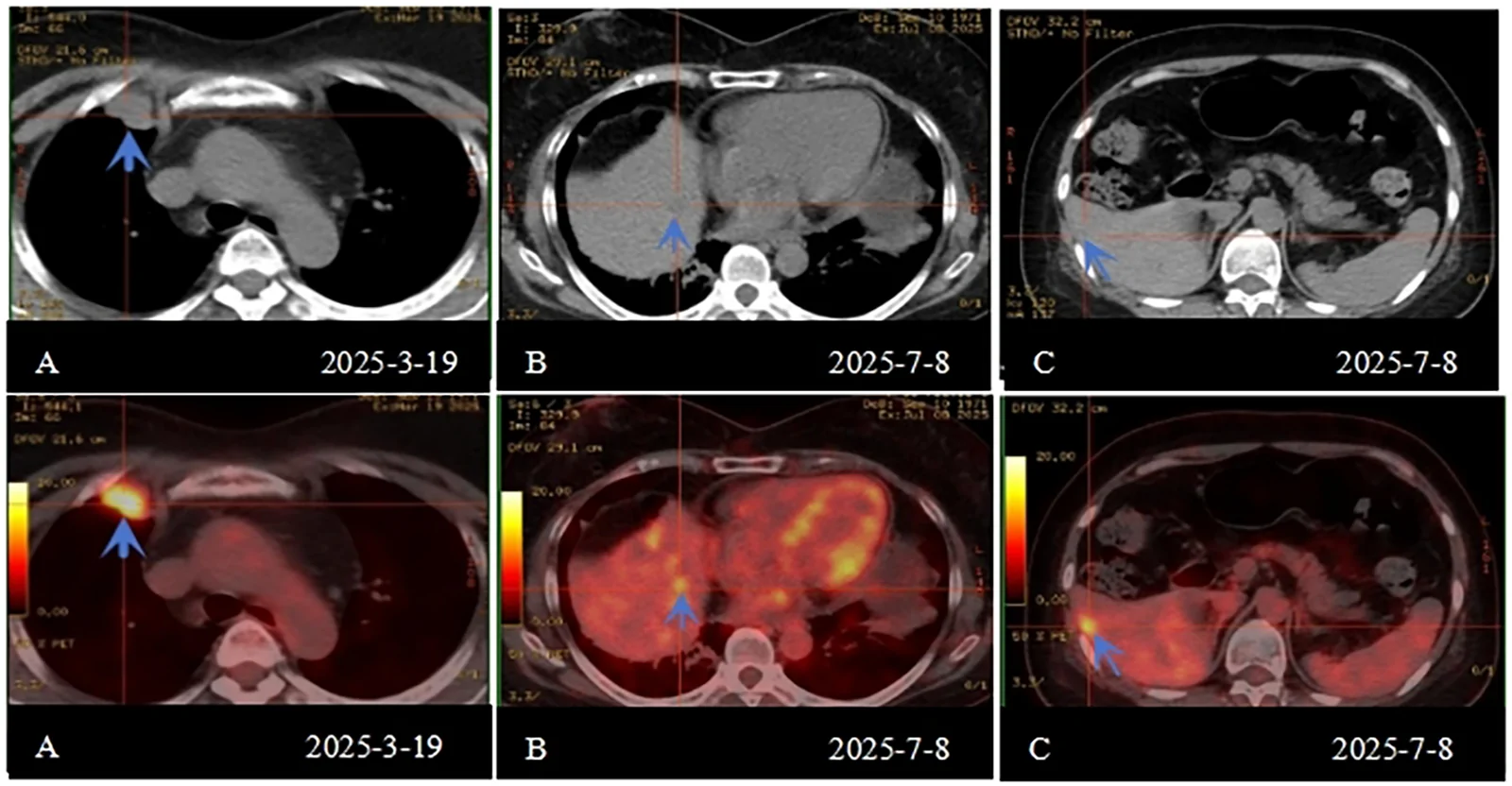
PET/CT imaging. **(A)** Repeat PET/CT imaging on March 19, 2025 demonstrates newly identified right pleural metastasis (blue arrow). **(B, C)** PET/CT revealing newly developed hepatic metastases (July 8, 2025): **(B)** Hepatic segment VI (S6) metastasis (blue arrow). **(C)** hepatic segment VIII (S8) metastasis (blue arrow).

**Figure 4:**
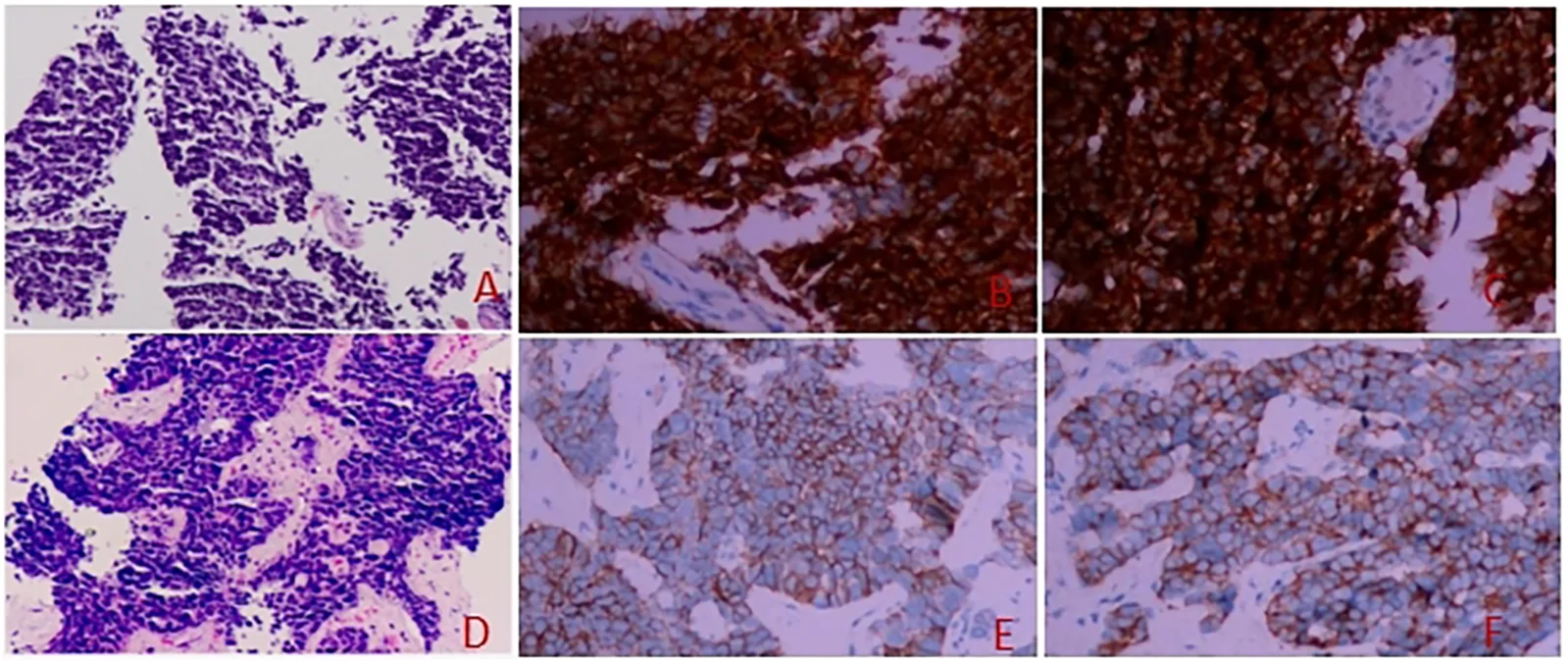
Pathological findings. **(A–C)** Right pleural lesion biopsy (March 2025): **(A)** HE staining, **(B)** CD56 (3+), **(C)** Syn (3+). **(D–F)** Hepatic lesion biopsy (July 2025): **(D)** HE staining, **(E)** CD56 (2+), **(F)** Syn (2+). (EnVision method, original magnification ×200).

**Figure 5:**
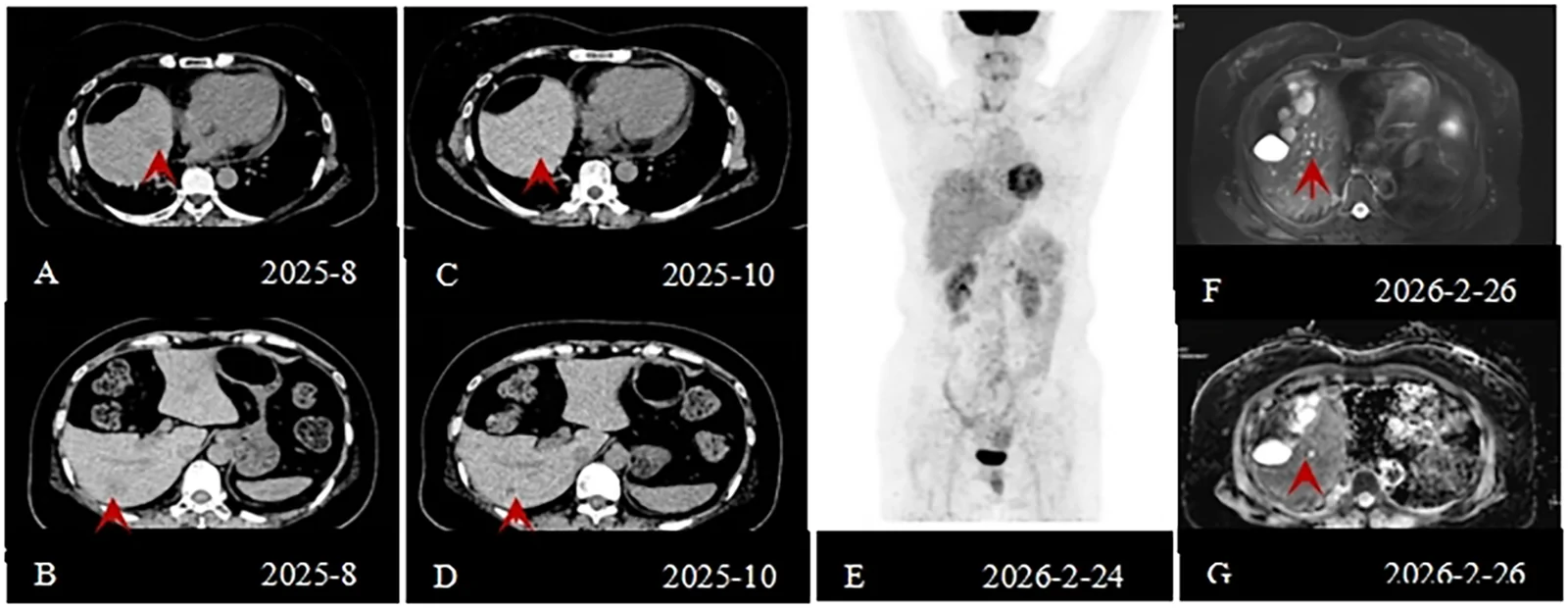
Changes in hepatic metastatic lesions before and after treatment. **(A, B)** Baseline imaging prior to osimertinib combined with EC regimen (August 2025; red arrows). **(C, D)** Imaging after 3 cycles of osimertinib combined with EC regimen (October 2025; red arrows). **(E)** PET/CT MIP image showing no abnormal hypermetabolic lesions in February 2026. **(F)** Hepatic segment VIII metastatic nodule (maximum diameter: 5 mm) on T2WI of non-contrast hepatic MRI in February 2026. **(G)** Hepatic segment VIII metastatic nodule (maximum diameter: 5 mm) on DWI of non-contrast hepatic MRI in February 2026.

**Figure 6:**
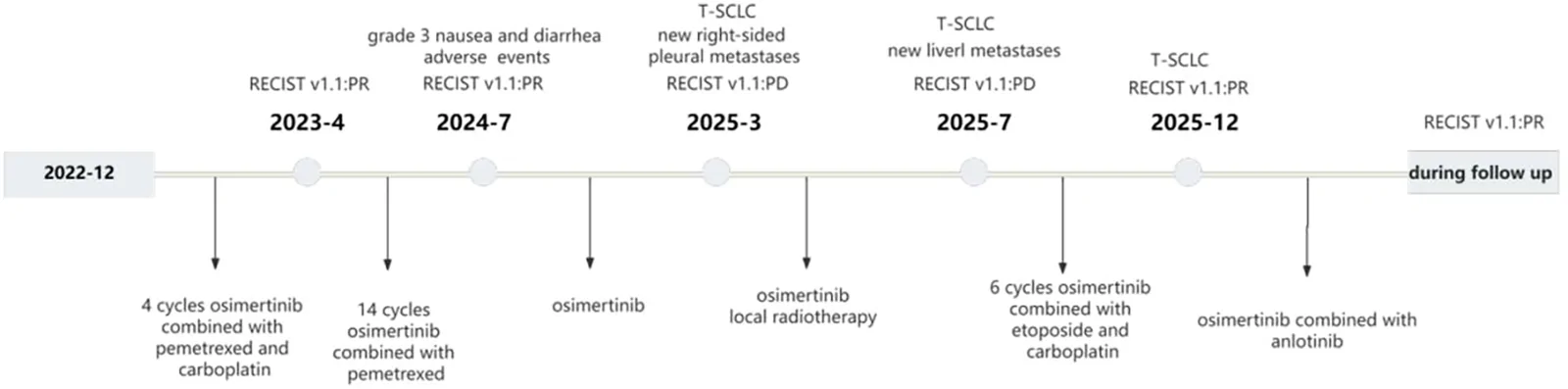
Treatment timeline and response assessment in the patient.

## Discussion

T-SCLC predominantly occurs in patients with lung adenocarcinoma harboring EGFR-TKI resistance mutations. The incidence is low and the transformation process is often insidious. Diagnosis relies on repeat biopsy. Histopathological examination remains the gold standard. However, because repeat biopsy is invasive, it is mainly performed in patients with EGFR-mutant lung adenocarcinoma who develop resistance to EGFR-TKI therapy. Its use in other lung cancer populations is limited. As a result, the true incidence of T-SCLC may be underestimated. After small cell transformation, the disease usually progresses rapidly and the prognosis is extremely poor. Treatment strategies also differ markedly from those used before transformation. Therefore, early identification of high-risk patients is essential to improve detection rates and optimize outcomes.

The mechanism of small cell transformation remains unclear. Currently, two main hypotheses are widely recognized. The first is the tumor heterogeneity hypothesis, which posits that both lung adenocarcinoma and small cell carcinoma pathological components coexist at initial diagnosis. However, due to limitations in pathological sampling techniques, small tissue biopsies cannot fully represent the overall tumor architecture (9). A retrospective study involving pathological re-examination of specimens from patients who underwent SCLC transformation revealed that small cell carcinoma components were already present prior to transformation (10). Nevertheless, most reports suggest that the tumor heterogeneity hypothesis cannot entirely account for the clinical reality (11). The second is the lineage plasticity hypothesis, which proposes that NSCLC and SCLC cells may originate from a common tumor stem cell population (12–14). Lung adenocarcinoma is derived from alveolar type II epithelial cells and Clara cells, whereas small cell lung cancer originates from neuroendocrine cells in the central airways. Further investigation demonstrated that type II alveolar epithelial cells possess the potential to transdifferentiate toward SCLC phenotype. Under sustained selective pressure from EGFR-TKI and other targeted agents, this lineage plasticity is promoted, facilitating SCLC transformation (15).

The molecular mechanisms underlying small-cell transformation remain incompletely defined. Converging evidence implicates multiple molecules and signaling pathways. Early events frequently involve alterations in *TP53* and *RB1* most commonly loss of function with concomitant *RB1/TP53* inactivation associated with a 43-fold increase in the risk of SCLC transformation (16). In NSCLC patients harboring concurrent *EGFR/RB1/TP53* mutations, the incidence of small-cell transformation after EGFR-TKI therapy is approximately 18%, sixfold higher than in patients without *RB1* and *TP53* inactivation (17). The PI3K/AKT pathway also appears to play a central role in neuroendocrine transdifferentiation (18, 19). Offin et al. reported *MYC* amplification in 7% of transforming SCLC cases with concurrent *EGFR/RB1/TP53* alterations (17), and experimental studies indicate that *MYC* activation is a key driver promoting transdifferentiation of type II alveolar epithelial cells toward an SCLC phenotype (20). *SOX* gene mutations have been identified in a subset of transforming SCLC with *EGFR/RB1/TP53* triple mutations. However, whether *SOX* genes directly participate in this process remains unclear (17). In the present case, initial genomic profiling revealed *EGFR/RB1/TP53* mutations, in line with previous reports. Taken together, these findings suggest that NSCLC patients with concurrent *EGFR*/*RB1*/*TP53* mutations are at high risk for small cell transformation. Such patients warrant close monitoring for possible evolution to small cell carcinoma.

Neuron-specific enolase (NSE) and ProGRP are serological biomarkers with high sensitivity and specificity for SCLC. However, they are not routinely measured in NSCLC. Previous studies have shown that NSE and ProGRP levels may rise before small cell transformation (21–23). In the present case, the patient had advanced EGFR-mutant lung adenocarcinoma with concurrent *TP53* and *RB1* mutations. Baseline genetic testing at diagnosis revealed *EGFR*/*RB1*/*TP53* triple mutations. This finding prompted close surveillance for small cell transformation, including regular monitoring of NSE and ProGRP. ProGRP levels began to increase about two months before histological confirmation of small cell transformation. At that time, imaging still showed stable disease, and the treatment regimen was not changed. After small cell transformation was confirmed and therapy was adjusted, ProGRP levels decreased in parallel with radiologic tumor regression. These observations suggest that NSE and ProGRP monitoring may be useful in high-risk patients. Rising levels of these markers should raise suspicion for small cell transformation and prompt timely repeat biopsy to confirm histologic conversion. Repeat biopsies are mostly invasive procedures. In lung cancer patients with malignant pleural effusion, percutaneous closed pleural brushing is a simple, minimally invasive technique for obtaining pathological specimens and may be considered as an alternative approach (24).

The patient carried an *EGFR L858R* mutation with a concurrent *TP53* mutation. Both are associated with poor prognosis. The patient also presented with a high tumor burden at diagnosis. The FLAURA2 trial showed that osimertinib plus chemotherapy provides a greater survival benefit than osimertinib alone (25). Based on these data, first-line therapy in this case was osimertinib combined with pemetrexed and carboplatin. *EGFR* mutations are rarely detected in primary SCLC. In contrast, repeat biopsy studies have shown that 84% of SCLC cases retain the same *EGFR* mutation profile as the original NSCLC (5). This finding suggests that most such SCLC cases arise through transformation from the primary NSCLC rather than as *de novo* SCLC. Regarding the mechanisms underlying transformation following EGFR-TKI resistance, two predominant hypotheses have been proposed: the combined lung cancer hypothesis and the common origin hypothesis (10, 20, 26). Based on these considerations, we conclude that the present case represents transformed small cell lung cancer. Currently, there are no standardized treatment guidelines for T-SCLC. At the time of histologic transformation, the right pleural lesion represented isolated oligoprogression, while the primary pulmonary lesion and previously identified osseous metastases remained radiographically controlled. Although platinum–etoposide would have been a conventional systemic option after histologic confirmation of T-SCLC, the MDT initially elected to continue osimertinib with local radiotherapy because progression was confined to a single pleural site while all other known disease remained controlled. This represented an individualized strategy rather than an established standard for transformed SCLC. However, multiple hepatic metastases developed three months later. Repeat biopsy of the hepatic lesions with molecular profiling continued to demonstrate T-SCLC with concurrent *EGFR* mutation. Pleural metastases demonstrated weak Rb immunoreactivity (1+), whereas the liver metastases were entirely Rb-negative. Loss of Rb protein expression is a common feature of SCLC and is consistent with disruption of the RB pathway; however, immunohistochemical loss of Rb should not be considered equivalent to a specific *RB1* genomic event. The change from weak Rb expression in the pleural lesion to complete loss in the hepatic lesion may reflect spatial and temporal tumor heterogeneity or clonal evolution; however, sampling and technical variability cannot be excluded. Multiple retrospective studies and case reports have demonstrated that etoposide plus platinum chemotherapy is the preferred treatment modality for T-SCLC (27, 28). Compared with chemotherapy alone, first-line treatment combining chemotherapy with EGFR-TKIs has been shown to prolong progression-free survival (PFS) (4, 11, 17, 29). Therefore, following the development of hepatic metastases, we adjusted the treatment regimen to osimertinib targeted therapy combined with EC chemotherapy. After six cycles of osimertinib combined with EC chemotherapy, the hepatic tumors exhibited regression, with imaging evaluation demonstrating PR. Multiple studies have indicated that despite achieving relatively high objective response rates with etoposide plus platinum chemotherapy (4, 5), PFS remains short, with a median PFS of 3.5 months (4, 7), and disease progression typically occurs within several months (11). Prognosis is even worse if small cell transformation is not detected in a timely manner. How to prolong survival in these patients remains a therapeutic challenge. Retrospective studies have shown that the combination of anlotinib with chemotherapy in T-SCLC confers longer overall survival compared to regimens without anlotinib (11). Case reports indicate that targeted therapy with osimertinib combined with anlotinib has demonstrated preliminary efficacy in transformed SCLC (30). In recent years, multiple novel agents targeting delta-like ligand 3 (DLL3) have shown promising potential in the treatment of SCLC. In the study by Saalfeld et al, a rationale for the use of DLL3-targeted therapies in transformed SCLC was established by identifying DLL3 as a relevant therapeutic target (31). Bcl-2 inhibitors have been proven to show limited clinical settings in T-SCLC (12). To extend the patient’s survival, we adjusted the treatment to osimertinib combined with anlotinib as maintenance therapy following completion of six cycles of EC chemotherapy. Currently, the tumor remains stable, and both PFS and OS have exceeded the median values reported in the literature.

This study has several limitations. First, it is a single-case report with an extremely small sample size. The conclusions are based on individualized clinical observations and cannot be generalized to all patients with small-cell transformation of EGFR-mutated lung adenocarcinoma. In addition, molecular testing was performed using different specimen types and gene panels at the three time points, precluding direct quantitative comparison of variant allele frequencies across assays. At the time of pleural transformation, genomic testing was performed using plasma rather than the pleural tumor tissue, which limited direct molecular correlation between the transformed histology and the genomic profile at that time point.

## Conclusion

In conclusion, clinicians should be vigilant for small cell lung cancer transformation in patients with EGFR-mutant lung adenocarcinoma undergoing EGFR-TKI therapy. For high-risk populations harboring concurrent *TP53* and *RB1* mutations, dynamic monitoring of tumor markers such as NSE and ProGRP may be considered. Timely detection of small cell transformation, followed by individualized multimodal treatment strategies tailored to the characteristics of the transformed tumor, may improve patient prognosis.

## Data Availability

The original contributions presented in the study are included in the article/supplementary material. Further inquiries can be directed to the corresponding authors.
